# Differential expression patterns of conserved miRNAs and isomiRs during Atlantic halibut development

**DOI:** 10.1186/1471-2164-13-11

**Published:** 2012-01-10

**Authors:** Teshome T Bizuayehu, Carlos FC Lanes, Tomasz Furmanek, Bård O Karlsen, Jorge MO Fernandes, Steinar D Johansen, Igor Babiak

**Affiliations:** 1 University of Nordland, Faculty of Biosciences and Aquaculture, Postbox 1490, 8049 Bodø, Norway; 2 University of Bergen, Department of Biomedicine, Postbox 7804, N-5020 Bergen, Norway; 3 University of Tromsø, Department of Medical Biology, Faculty of Health Sciences, 9037 Tromsø, Norway

## Abstract

**Background:**

MicroRNAs (miRNAs) play a major role in animal ontogenesis. Size variants of miRNAs, isomiRs, are observed along with the main miRNA types, but their origin and possible biological role are uncovered yet. Developmental profiles of miRNAs have been reported in few fish species only and, to our knowledge, differential expressions of isomiRs have not yet been shown during fish development. Atlantic halibut, *Hippoglossus hippoglossus *L., undergoes dramatic metamorphosis during early development from symmetrical pelagic larval stage to unsymmetrical flatfish. No data exist on role of miRNAs in halibut metamorphosis.

**Results:**

miRNA profiling using SOLiD deep sequencing technology revealed a total of 199 conserved, one novel antisense, and one miRNA* mature form. Digital expression profiles of selected miRNAs were validated using reverse transcription quantitative PCR. We found developmental transition-specific miRNA expression. Expression of some miRNA* exceeded the guide strand miRNA. We revealed that nucleotide truncations and/or additions at the 3' end of mature miRNAs resulted in size variants showing differential expression patterns during the development in a number of miRNA families. We confirmed the presence of isomiRs by cloning and Sanger sequencing. Also, we found inverse relationship between expression levels of sense/antisense miRNAs during halibut development.

**Conclusion:**

Developmental transitions during early development of Atlantic halibut are associated with expression of certain miRNA types. IsomiRs are abundant and often show differential expression during the development.

## Background

Atlantic halibut, *Hippoglossus hippoglossus *L., the largest flatfish of Atlantic Ocean, is a species of commercial interest to the aquaculture industry. Halibut's early developmental stages are prolonged and morphologically defined [1,2]. The critical developmental stages, when dramatic changes in signaling, physiology and morphology occur, include: (i) maternal to zygote transition (MZT), when maternally stocked transcripts are degraded and zygote transcripts take control over the development; (ii) organogenesis, when the germ layers are formed; (iii) hatching, when the embryo becomes a free-swimming larva; (iv) first feeding, when active movement, visualization, recognition of prey, and exogenous feeding begin; and (v) metamorphosis, the most dramatic morphological and behavioral change in a flatfish during the transition from a symmetric post-larval to an asymmetric juvenile stage, when migration of one eye towards the other one occurs across the skull [1].

MiRNAs are small (18 - 26 nucleotides) non-coding RNAs that control and regulate target genes, mostly in a suppressive way [3,4]. In vertebrates, miRNAs are important in various developmental processes including MZT, maintenance of cell and tissue identity, organ development, hematopoietic lineage modulation, adipocyte differentiation, and imprinting [5]. Developmental profiles of miRNAs have been reported in few fish species only, including model species such as zebrafish (*Danio rerio*), fugu (*Takifugu rubripes*), tetraodon (*Tetraodon nigroviridis*), and medaka (*Oryzias latipes*). Recently, miRNAs have been characterized in Atlantic cod (*Gadus morhua*), Asian seabass (*Lates calcarifer*) and Japanese flounder (*Paralichthys olivaceus*) [6-10]. Functional studies have been performed only on zebrafish and medaka [11-14]. These studies showed the conservation of miRNA sequences among distantly related teleosts and variations in the expression pattern of selected miRNAs during the development [14], and indicated that miRNA sequence conservation did not reflect spatial or temporal conservation of expression [15]. Some species-specific miRNAs have been found [6,16].

IsomiRs are size variants of miRNAs. Their occurrence has been observed along with the main miRNA types [17,18], but their origin and possible biological role are not known yet. Modifications and size variations have been reported during miRNA maturation in different organisms [19-24]. These alterations have been suggested to influence stability, effectiveness, protection or degradation of mature miRNAs [19-23,25]. Imperfect cleavage of pri- and/or pre-miRNAs by Drosha and Dicer, respectively, affects mature miRNAs size and indicates that size heterogeneity could be an enrichment strategy of miRNAs for target diversification [24]. IsomiRs can play important roles under both physiological and disease conditions [17,26,27]. To our knowledge, differential expressions of isomiRs have not yet been shown during ray-finned fish development.

Various approaches have been used to characterize and profile miRNAs with or/and without prior knowledge of sequences. The SOLiD high throughput sequencing platform has been used to identify and characterize small RNAs in various species [8,28,29]. The depth of coverage from SOLiD platform is a considerable advantage over other comparable platforms, such as the 454 pyrosequencing platform [8]. We applied high-throughput SOLiD sequencing to identify and profile Atlantic halibut miRNAs during the development. We discovered putative novel miRNAs and isomiRs, and demonstrated that isomiRs were differentially expressed during halibut development.

## Results

### Sequence statistics and miRNA identification

**Figure 1:**
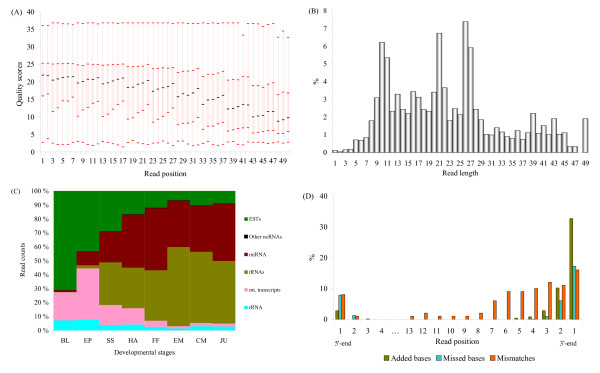
**Read statistics for deep sequencing of Atlantic halibut small non-coding RNA**. A) Quality score of reads averaged at each position for all samples; the box indicates inter-quartile range, where the lower boundary shows 1^st ^quartile and the top boundary shows the 3^rd ^quartile with the median line in between. B) Read length distribution after removal of adaptor sequences. The three peaks of read size frequencies represent very small RNA, miRNA/siRNA, and piRNA (see details in Results). C) Proportion of reads mapped to different databases excluding unmapped reads; other non-coding RNAs are < 1% in all stages. BL, EP, SS, HA, FF, EM, CM, and JU stand for blastula, epiboly, somitogenesis, hatching, first feeding, early metamorphosis, climax metamorphosis and juvenile developmental stages, respectively. D) Overall average mismatches, missed and added bases in halibut mature miRNAs compared to mature miRBase reference sequences.

The last 15 color reads from the 3' end, which were poor in quality, were removed from all tags (Figure 1A). This pre-processing did not affect the actual size of miRNAs. About 72 million reads were obtained from eight developmental stages of Atlantic halibut. Out of it, 70.7% were longer than 15 nucleotides, nt (Additional File 1). Length distribution of all reads showed three peaks (Figure 1B), with the majority (54.5%) being between 16 and 26 nt long. Reads longer than 15 nt were composed of 2.2% mitochondrial transcripts, 8.2% tRNA, and 0.8% rRNA sequences. The size distribution of reads that mapped to different databases is shown in Additional File 2. On average, 7.2% of these reads were mapped to known miRNAs in all examined developmental stages with the highest percentage (14.9%) in the first feeding stage and the lowest (0.2%) in the blastula stage. In addition, 4.5 and 0.16% of the reads were mapped to halibut ESTs and to other non-coding RNAs, respectively (Figure 1C). Remaining reads were unmapped.

Most of conserved fish miRNA families annotated in miRBase release version 16 were identified in the present study, with except for miR-208 and miR-155 families. The identified families consisted of 199 types of conserved miRNAs (Additional File 3). miRNA diversity was increasing along with the developmental advancement (Additional File 4).

We found extensive length variation in several miRNAs (Figure 1D and Additional File 5). These modifications were more pronounced at the 3' end, where 46% of miRNAs had added bases and 24% had missed bases compared to the reference sequences. Likewise, the majority (67%) of mismatches was observed on the last six nucleotides from 3' end of mature miRNAs, but 8% of the miRNAs showed a mismatch at the first base of the 5' end.

### Expression profile of conserved miRNAs

**Figure 2:**
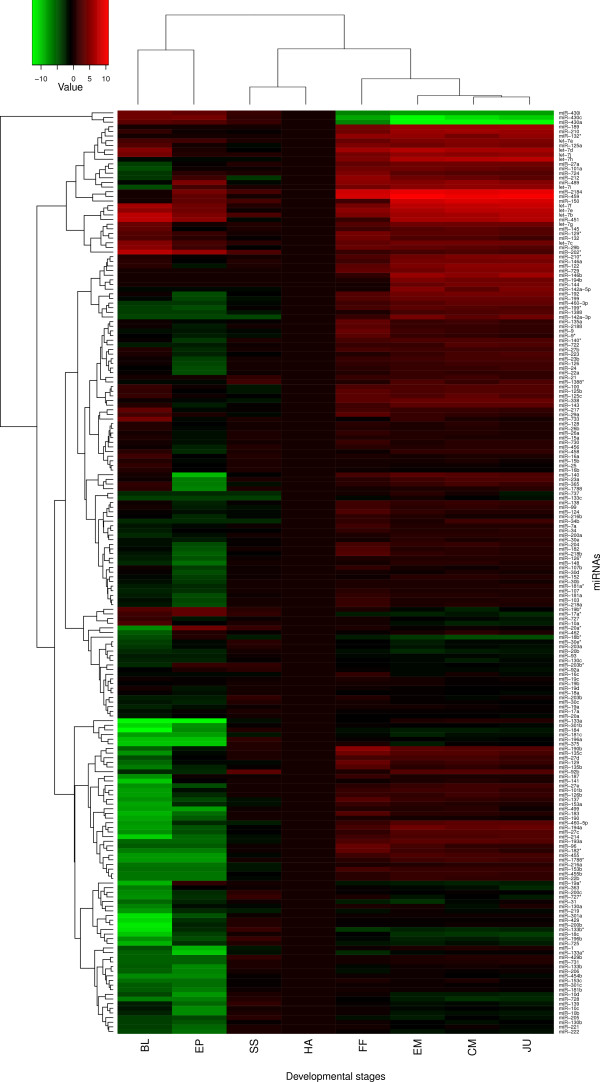
**The heatmap of identified miRNAs during Atlantic halibut early ontogenesis (see Figure 1C legend for abbreviations)**. The heatmap is obtained using R program version 2.12.0 (Euclidean distance, average linkage algorithm). There are 199 rows and 8 columns corresponding to each miRNA and developmental stage, respectively. The heatmap was drawn on log 2 normalized read counts in relation to the expression at hatching stage as a reference (no color), and downregulation and upregulation were denoted with shades of green and red, respectively.

Expression patterns of some miRNA families were associated with certain developmental stages (Figure 2). Hierarchical clustering showed four major expression patterns: a) early embryonic expression, which might be related to MZT and post-MZT clearance of maternal transcripts; b) embryonic expression, during organogenesis and hatching; c) feeding larvae expression, when larvae undertook exogenous feeding; and d) metamorphosis expression, during larva-to-juvenile transition.

**Figure 3:**
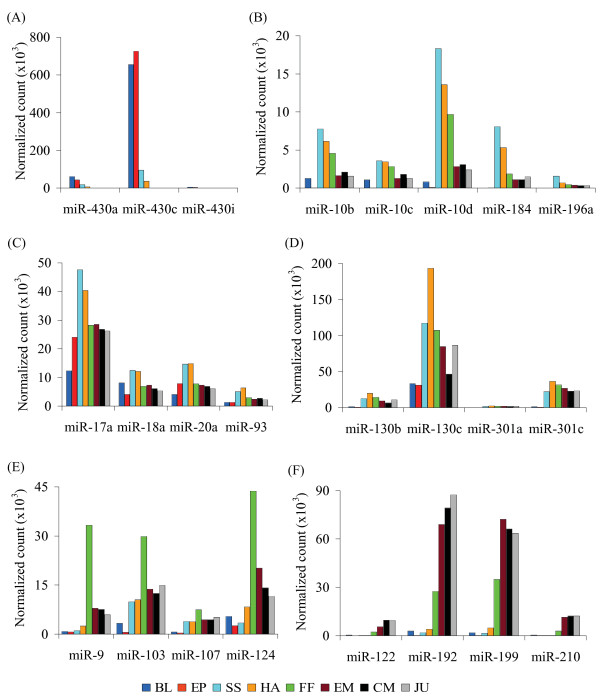
**Normalized read counts of selected miRNAs during eight stages of Atlantic halibut development**. See Figure 1C legend for specification of developmental stages.

**Figure 4:**
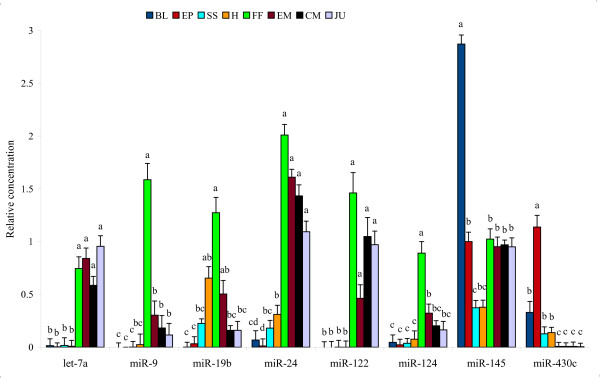
**Relative expression of selected miRNAs using RT-qPCR**. Different letters indicate statistically significant differences (P < 0.05) between means. Error bar is S.E.

miRNA diversity and expression intensity varied among the developmental stages. At early stages (blastulation and gastrulation), fewer types of miRNAs were found than in later stages (Additional File 4). The most dominant miRNAs at earlier stages belonged to the miR-430 family, showing the highest expression during the gastrulation and ceasing after hatching (Figures 3A and 4).

Among miRNAs involved in the pattern formation and maintenance of cellular and tissue identity, miR-10, miR-17, miR-184, or miR-196 families were relatively highly expressed throughout somitogenesis and hatching (Figures 2, 3B and 3C), and miR-130 family was highly expressed around hatching stage (Figure 3D). Other miRNAs, such as miR-9, miR-103 family, miR-107 and miR-124 were expressed intensively during the first feeding stage (Figures 3E and 4).

Peak of expression of a number of miRNAs, such as miR-122, miR-192, miR-199, miR-206 and miR-210 was associated with specific developmental transition points (Figures 2, 3F and 4; Additional Files 3 and 4).

**Figure 5:**
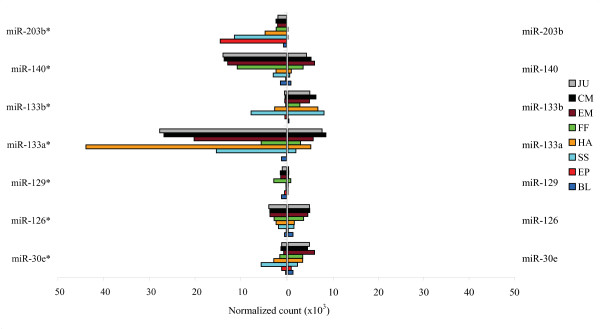
**Relative counts per million for guide and passenger strand miRNAs**. See Figure 1C legend for developmental stages label descriptions.

In some cases the passenger strand miRNAs were highly expressed compared to the guide strand. For example, miR-133a* and miR-140* had at least three-fold higher expression compared to their counterparts miR-133 and miR-140, respectively (Figure 5). At somitogenesis, the expression of miR-133b* was comparable to that of the guide strand, whereas miR-30e* expression at this stage was two times higher than the expression of the guide strand. However, in the later stages, the expression of miR-30e* and miR-133b* was dissipated comparing to their counterparts miR-30e and miR-133b, respectively. In addition, the expression of miR-203b* was higher than miR-203b in all examined stages of the development (Figure 5).

Our digital expression profile results were positively validated by RT-qPCR assay (*r *= 0.90, 0.99, 0.62, 0.72, 0.65, 0.99, 0.77 and 0.85 for let-7a, miR-9, mir-19b, miR-24, mir-122, miR-124, miR-145, and miR-430c, respectively; Figure 4).

### Differential expression of isomiRs during halibut development

**Figure 6:**
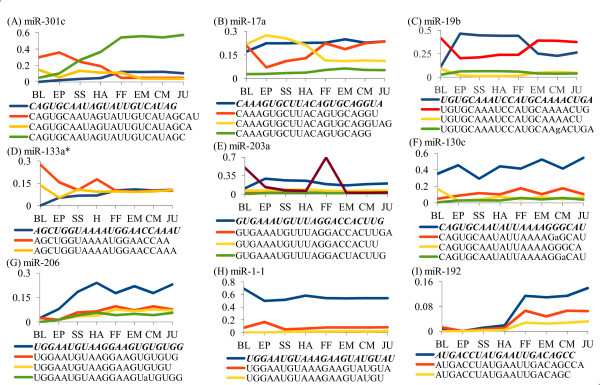
**IsomiRs expression pattern for selected miRNAs through early developmental stages of Atlantic halibut**. The reference sequences are in bold italic. Y-axis is a relative frequency. Developmental stages are specified in Figure 1C legend

Some miRNAs with up to three missed or added nts from the 3' end were expressed differently compared to full-sized miRNAs (Figure 6A-I). We found that the expression of isomiRs varied and some isomiRs showed distinct quantitative patterns throughout the development. This pattern was clearly visible in miR-301c, where single nt-added isomiR showed constant increase in the expression from blastula stage towards the juvenile stage, in contrast to the three nts-added isomiR in which expression declined during the development (Figure 6A). Also, single nt-missed and single nt-added isomiRs of miR-17a showed the same expression level at blastula stage, but in the later developmental stages they followed opposite trends (Figure 6B). Truncated miR-19b isomiRs showed a reciprocal expression pattern throughout the development compared to the non-truncated counterpart (Figure 6C). Stage-specific expression pattern of truncated isomiRs was observed in miR-203a (Figure 6E) and differential expression was observed in other tested isomiRs (Figure 6F-I). Although most of the added nucleotides were template nucleotides, there were non-template nucleotides in some of the isomiRs, such as miR-1-1, miR-125a, miR-203a, miR-206, and miR-375 (Additional File 5). The prominent non-template nucleotides were uracil or adenine, reaching 11.8% and 5.3% of number of reads in miR-1 and miR-375, respectively. Guanine additions were below 1% of reads, and cytosine additions were less than 0.01% (Additional File 5). Single nt-additions at 5' end were frequent in miR-204 (10.7%), miR-214 (22.4%), miR-22a (6.9%), and miR-455 (19.5%). Single nt additions at 3' end exceeded 60% frequency, in a number of miRNAs, such as miR-10b, miR-30c miR-126a, miR-135b, and miR-430d (Additional File 5).

We performed cloning and Sanger sequencing to verify the presence of isomiRs. Fifty out of 234 sequenced clones had miRNAs inserts. These sequences aligned to 30 different types of miRNAs. Clones containing miR-17 sequence showed size variations, including three nts truncation and addition at both 5' and 3' ends. Untemplated adenine at the 3' end of miR-17 was found. miR-206 clone sequences were truncated by 2 nts from the 3' end (Additional File 6).

### Identified novel miRNAs

**Figure 7:**
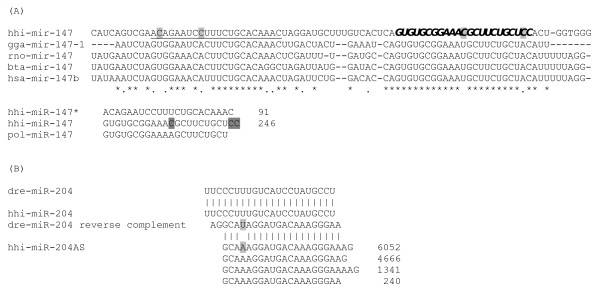
**A) Atlantic halibut mir-147 precursor sequence in comparison with human, rat, chicken, and cattle precursor sequences**. B) Alignment and sequence of novel antisense miRNA in Atlantic halibut. Mature guiding strand miRNA sequences are indicated in italic and bold, while miRNA* sequences are underlined. Differences in nucleotide among species are highlighted gray. Star (*) indicates the conservation among all species, whereas dot (.) indicates partial conservation. Numbers on the right indicate overall SOLiD read counts for the respective sequence.

We identified one precursor miRNA from EST dataset (Genebank ID: EB030760.1) having sequence similarity with mammalian and bird miR-147. We also found reads of a putative novel miR-147* sequence in the deep sequencing dataset (Figure 7A).

Antisense miRNA of reverse complementarity to miR-204 with one mismatch and additional nucleotides at the 3' end was another putative novel miRNA (Figure 7B). The expression pattern of the antisense (miR-204AS) throughout the development was different from that of the miR-204. The ratio antisense:sense expression was the highest at the blastula stage, then declined as the development progressed. Sense miRNA expression was higher than antisense from the first feeding stage onwards (Table 1).

**Table 1:** The ratio between antisense and sense strands expression of miR-204 during Atlantic halibut development.

*Developmental stages*	*Anti-sense:sense ratio*
Blastula	872.5
Epiboly	195.42
Somites	4.78
Hatching	1.41
First feeding	0.07
Early metamorphosis	0.2
Climax metamorphosis	0.35
Juvenile	0.49

## Discussion

### Characterization Atlantic halibut small RNAs

The three distinct peaks in the size distribution of Atlantic halibut small RNA consisted of 26 - 27 nts cluster corresponding to piwi-interacting RNAs, 20 - 22 nts cluster containing miRNAs and short interfering RNAs, and 10-11 nts cluster of unknown origin and function (Figure 1B). To our knowledge, these very short RNAs have not been characterized yet; previously, they were considered as degradation products and removed from the datasets during the data processing [30,31].

The number of reads in size range between 16 and 26 nts was high (54.5%) indicating that the libraries were enriched with miRNAs. miRNAs are classified into different families based on their seed sequence similarity that span 2 - 8 nucleotide positions from 5' end of mature miRNA [6]. This conservation allows comparison among species. In the present study, all but two known fish miRNA families were identified, confirming high conservation of miRNAs among teleosts.

### Expression profile of conserved miRNAs and halibut development

Atlantic halibut has morphologically defined developmental stages that can be inferred along with the expression of various genes [32-35]. The increase in miRNA diversity along with the developmental advancement (Additional File 4) indicates the importance of miRNAs in the tissue and organ differentiation, similar to mice [36], chicken [37], other fish [38,39], and invertebrates [40]. It has been demonstrated that miRNAs play pivotal roles in the developmental progression and maintenance of tissue identity in several species [11,39,41-44]. In the present study, miRNAs were expressed either uniquely or ubiquitously during the development (Figure 2).

Maternal-zygotic transition (MZT) was the earliest developmental transition investigated in the present study. In mammals, the onset of MZT occurs during the three first cell divisions [45], while in most teleosts it takes place around the beginning of gastrulation [46]. Maternal transcripts clearance and MZT in zebrafish and medaka is associated with the expression of miR-430 family [11,12,39,47]. Similarly, we demonstrated previously that maternal transcripts decline around MZT in Atlantic halibut [48]. High expression of miR-430 at the onset of gastrulation (Figures 2 and 4) suggests that MZT in Atlantic halibut is facilitated by miRNAs.

We found that miR-10, miR-17, miR-184, or miR-196 families were relatively highly expressed throughout somitogenesis and hatching (Figures 2, 3B, C). miR-17 family is involved in early development and functional cell differentiation in mammals [49,50]. High expressions of miR-17 family during halibut organogenesis can imply this assertion. miR-10 and miR-196 have been identified along with *Hox *genes cluster in fruit fly, zebrafish, pufferfish, mouse, and human [51,52]. miR-130 family, highly expressed around halibut hatching stage (Figure 3D), have been implicated in regulation of angiogenesis [53]. Thus, the miR-130 family could also promote development of the blood and lymphatic vascular systems in halibut.

Some miRNAs, such as miR-9, miR-103 family, miR-107, and miR-124 were expressed intensively during the first feeding stage (Figures 3e and 4). Previous studies have shown exclusive expression of some miRNAs in certain tissue types [47,54]. For example, in zebrafish, miR-9 is expressed in differentiated neurons, and miR-124 is broadly expressed in both proliferative and differentiated brain cells [47,54]. The expression of miR-9 and miR-124 in halibut was synchronized with the beginning of exogenous feeding, which requires intensified neural functions for active movement, identification, and capturing of a prey. Also, miR-103 was expressed in similar fashion during the first feeding. miR-103 is involved in intestinal cell proliferation in mouse, [55], and in zebrafish it is expressed in the gut [39]. High expression of miR-103 during the start of exogenous feeding in halibut might be related to intestinal growth.

Various miRNAs, including miR-192 and miR-199, are differentially expressed during metamorphosis of Japanese flounder [9]. In the present study, miR-122, miR-192, miR-199 and miR-210 were among miRNAs highly expressed during halibut metamorphosis (Figures 2 and 4). Erythropoiesis is regulated by many miRNAs including miR-210 [56-58]. Specific expression of a number of miRNAs during halibut metamorphosis indicates the possible involvement of miRNAs in this developmental transition.

Relatively high expression of miRNA*'s have been reported during zebrafish development [39] and in Japanese flounder [9]. Our results support these findings. In a number of cases halibut miRNA* expression exceeded miRNA expression and showed development-specific pattern (Figure 5). miRNA*s can be part of the post-transcriptional regulatory network during the development [59].

### IsomiR expression during halibut development

Size variants found in multiple miRNAs during Atlantic halibut development (Figure 6 and Additional File 5) raise the question whether they play any regulatory role. The presence of truncated or added template nucleotides in 89 out of 199 miRNAs in this study is similar to that reported in few other species [18,60].

Frequent 5' nucleotide addition observed in some of the miRNAs (Additional File 5) might be explained as imperfect cleavage of pri- and/or pre-miRNAs by Drosha and Dicer micro processors. Starega-Roslan et al. [24] have shown that the structure of hairpins including the symmetry of internal loops and number of nucleotides on bulges affects size heterogeneity of mature miRNAs in human cell lines. Other possible explanation is that miRNA size variants can result from paralogue genes [26], or different allelic forms.

A number of reads in the present study showed significant additions of A or U. The presence of non-template nucleotides at the 3' end in our datasets (Additional file 5) may indicate post-maturation modifications of miRNAs, as shown in mammals and insects [16,18,26,61,62]. miRNA modifications including 3'-methylation and adenosine-to-inosine editing have been reported in several organisms [19-23]. These changes are implicated in the stability, protection and destruction of miRNAs, as well as in determining miRNAs efficiency in loading to Argonaute protein complex [16,25,63,64].

We found isomiRs in both cloning and next generation sequencing datasets; for example, miR-17a, miR-206, and miR-204 had identical isomiRs (Additional Files 5 and 6). Similar results have been reported in different organisms and platforms [26,27,60]. All those data suggest that end modifications in mature miRNA could unlikely be a processing error.

Differential expression of isomiRs has recently been reported during *Drosophila *development [18]. Also, it has been shown that both 5' and 3' truncated versions of miRNAs were incorporated in RNA-induced silencing complex (RISC) [27], indicating possible functional role of isomiRs. We demonstrated that isomiRs showed clearly distinctive expression patterns during the development of halibut (Figure 6). Our data suggest possible diversification strategy for selective exclusion/inclusion of certain target genes from/for repressive control during the specific stages of development. Therefore, we hypothesize that isomiRs can be legitimate and biologically meaningful forms of mature miRNAs, which deserve further investigation.

### Novel miRNAs in Atlantic halibut

Discovery of species-specific miRNAs largely depend upon the availability of genomic resources, since mapping and extracting precursor miRNA sequences is a crucial step of the process. Given the limitation of adequate genome resources for Atlantic halibut, we identified only single precursor miRNA from Atlantic halibut EST data using stringent criteria. Although this precursor miRNA was characterized in mammals and chicken [6,26,36,65,66], the miRNA* sequences have not been characterized yet [6]. The other novel miRNA was antisense miRNA (Figure 7B). Transcription of miRNA iab-4 locus from both DNA strands shows developmental importance of sense/antisense miRNAs in flies as well as in mammals [67]. Deep sequencing data revealed miRNA sense/antisense expression during silkworm development [30]. The gradual shift between the antisense and sense miRNA expression during the Atlantic halibut development suggests that antisense miRNAs can have a functional role.

## Conclusions

We have identified 199 conserved miRNAs, one miRNA*, and one novel antisense miRNA during Atlantic halibut ontogenesis. We profiled their expression pattern using SOLiD deep sequencing technology and verified it using RT-qPCR. We demonstrated differential expression of miRNAs throughout major developmental transitions. Some miRNA*'s showed higher expression than their miRNA counterparts. Also, we found reciprocal expression levels of sense/antisense miRNAs during halibut development. We demonstrated differential expression patterns in a number of isomiRs. Whether isomiRs are biologically meaningful it will be addressed in the future investigations.

## Methods

### Experimental animals

Fish were treated and samples were obtained following the procedures for animal experimentation provided by National Animal Research Authority in Norway. Gametes were obtained from broodstock fish held at the Marine Research Station of the University of Nordland, Bodø, Norway. In total, eggs from 7 females were fertilized and incubated in filtered 33‰ sea water following the procedures described by Babiak et al. [68]. Briefly, eggs from each individual female were incubated separately in flow-through conical 280 l tanks at 5.0 ± 0.5 ^°^C until hatching. Larvae were then transferred to conical 500 l tanks for first feeding and water temperature was gradually increased to 12 ± 1°C. Larvae were fed *Artemia salina *strain Franciscana (Salt Creek Inc, Utah, USA), while weaned larvae and juveniles were fed formulated dry-feed (Gemma, Skretting, Norway) *ad libitum*.

### Sampling and RNA extraction

Atlantic halibut embryos from each female progeny group were collected at the following developmental stages: blastula (2 days post fertilization, dpf), 25% epiboly (4 dpf), 30 somites (11 dpf), hatching (15 dpf), first feeding (~46 days post hatching, dph), early metamorphosis (~61 dph), climax metamorphosis (~92 dph), and juvenile (~112 dph). Halibut developmental staging was based on Pittman et al. [69] and Sæle et al. [1]. Total RNA was extracted from fresh or snap-frozen in liquid nitrogen samples using TRIzol Reagent (Invitrogen, Oslo, Norway). Total RNA integrity and quantity were checked using Bioanalyzer (Agilent Technologies, Waldbronn, Germany).

### Small RNAs library preparation and sequencing

Libraries of small RNAs were prepared by the SOLiD™ small RNA expression kit, SREK (Applied Biosystems, Austin, Texas, USA) in accordance with the manufacturer's recommendations. In brief, Total RNAs were mixed with adapter mix A for 16 h ligation, which allowed sequencing from the 5' end of small RNAs, followed by reverse transcription and RNase H treatment. Libraries of cDNA were prepared using barcoded 3' primers for downstream sample multiplexing. Based on a trial PCR, large scale PCRs were performed with the following PCR cycle adjustments: 18 cycles for blastula stage, 17 cycles for epiboly stage, 16 cycles for hatching stage and 15 cycles for the remaining samples. These adaptor-ligated PCR products were then size-selected (105 - 150 bp) using 6% PAGE gel (Invitrogen), quantified using a Bioanalyzer (Agilent Technologies) and standardized to obtain DNA libraries that were suitable for emulsion PCR. Samples were run on half a slide in two quadrants with 50 nucleotides read length on the SOLiD 3 system (Applied Biosystems).

### Cloning of miRNAs

Small RNA constituents of pooled RNAs from the eight developmental stages were recovered using flashPAGE fractionators (Ambion, Austin, Texas, USA) following the manufacturer's protocol. The recovered small RNA was ligated to adapter mix A of small RNA expression kit and reverse transcribed. cDNA was PCR amplified under the following thermal cycle conditions: initial denaturation of 95°C for 5 min, followed by 18 cycles of 95°C for 30 s, 62°C for 30 s, and 72°C for 30 s, and final extension of 72°C for 7 min. The product was run on 6% native PAGE and the product within the size range 105 - 150 bp was excised and eluted from the gel. The eluted product was cloned into psTBlue-1 AccepTor Vector (Novagen, Darmstadt, Germany) and transformed to NovaBlue Singles Competent Cells (Novagen). Positive colonies were collected and PCR was performed with T7 (5'-CTAATACGACTCACTATAGGG) and sp6 (5'-TCTATAGTGTCACCTAAAT) primers using 35 cycles of 94°C for 1 min, 55°C for 1 min, and 72°C for 2 min, and final extension of 72°C for 5 min. Positive clones were sequenced using T7 primer.

### Identification of conserved and novel miRNAs

The trimmed reads were analyzed using the RNA2map (version 0.5.0) pipeline (Applied Biosystems Community Software). In short, reads corresponding to tRNA, rRNA, mtRNA, and adaptor sequences were filtered out using 25 seed length and 2 color mismatches. The remaining reads were mapped against Pisces (zebrafish, fugu, and tetraodon) and human miRBase release 16 [6] using 20 seed length and 2 color mismatches. The remaining reads were mapped against Atlantic halibut ESTs. All forward mapped reads with size in between 17 and 26 nts were considered as legitimate miRNAs, if they had five and above counts at least in one of the investigated developmental stages. Known miRNAs were further analyzed for length and nucleotide composition. Each miRNA was aligned separately and checked for nucleotide composition and missed bases. All truncated miRNAs with more than 5 missed bases from 3'end and more than 1 missed base from 5' end were removed from the dataset.

Since Atlantic halibut genome has not been sequenced yet, our approach to identify novel miRNAs was through homologous search against miRNA databases by allowing two nucleotides mismatches inside the seed sequences and considering the reverse complementary sequences as well as checking for miRNA* sequences that were not characterized. Reads were considered as legitimate novel mature miRNAs when mismatches were found within seed sequences and had above 1000 read counts in total.

The other approach to find novel miRNAs was computational prediction from Atlantic halibut ESTs and GSS downloaded from NCBI (http://www.ncbi.nlm.nih.gov/sites/entrez). We used stringent criteria to avoid false positive by putting multilayer screening. Briefly, vector sequences from EST data were removed and clustered by TGICL [70]. The expected precursor sequences were extracted from the EST and GSS sequences using srnaloop [71] under similar criteria as in Li et al. [72] with some modifications. The modifications were done based on fish precursor miRNA in miRBase, which showed average GC % in between 30% and 70%, average length 86 with range between 62 - 132 nts, and mfe < -25 kcal/mol. Further filtration steps were deployed using MiPred, which uses mfe and machine learning methods to discriminate real miRNAs from random miRNAs [73]. Only real precursor miRNAs were fetched from MiPred results. Each sequence was checked manually and sequence containing ambiguous bases, stretches of nucleotides and repeats were removed. Then candidate precursor miRNAs from EST were blasted against candidate precursor miRNA from GSS to filter out redundancies. Non redundant candidate miRNA precursors were blasted against miRBase stem-loop sequences database (downloaded from miRBase release 16). A blast result was classified as a legitimate known candidate precursor miRNA when a query sequence were mapped with single mismatch in 14 nucleotides and more than 80% of its length was covered by the alignment with e-value < 0.1. Sequences without significant matches were used as putative precursor miRNAs. miRBase unmapped reads were mapped to these sequences using RNA2map with the same parameters as above. If sequences were mapped at the same positions outside the big loop region with more than 50 reads, then the putative precursor sequences were considered as legitimate miRNA precursors.

### Read count normalization and isomiRs expression

Numbers of reads were normalized using relative frequency of reads, that is, specific number of miRNA reads in a sample was divided by a total number of miRNA reads in the sample [74]. To be able to differentiate the expression of each miRNA and categorize them according to their expression pattern, a heatmap chart was drawn by transforming the normalized data to log 2 scales for visualization purpose. Hierarchical clustering with Euclidean distance and average linkage algorithm was performed using R-program version 2.12.0 [75].

Nine miRNAs with the highest read count were selected and analyzed for digital expression pattern of their isomiRs. IsomiRs were considered for this analysis only when they had above 1,000 reads with the relative frequency higher than 0.05 at least in one of the developmental stages. The relative frequency of each isomiR was calculated by dividing the number of reads of an isomiR in a sample by total number of reads of the miRNA.

### Reverse transcription quantitative polymerase chain reaction (RT-qPCR)

RT-qPCR was used to validate the digital expression profile of selected miRNAs (let-7a, miR-9, miR-19b, miR-24, miR-122, miR-124, miR-145 and miR-430c) using the TaqMan^® ^MicroRNA assay (Applied Biosystems, Foster City, Ca, USA) following the manufacturer's protocol. We used two technical and four biological replicates. Total RNA of each biological replicate was extracted from 8 embryos, 5 larvae and a juvenile. The reaction was performed on Light Cycler 480^® ^(Roche Applied Science, Rotkreuz, Switzerland) using white 96 well plates with thermal cycle conditions of 95°C for 10 min, followed by 45 cycles of 95°C for 15 s and 60°C for 1 min.

The expression values were calculated based on Cq values obtained from the light cycler with detection cut-off of 40 PCR cycles. Relative quantification was performed following Pfaffl's mathematical model [76]. The relative concentration of each sample was normalized according to Mestdagh et al. [77] using 5S RNA and U6 snoRNA as references. To meet the assumptions of parametric statistics, the data were cubic root transformed and statistical differences were computed with one-way ANOVA, followed by Tukey's HSD multiple comparison *post-hoc *test. *P *values of < 0.05 were considered as significant. Correlation between digital expression profiles and RT-qPCR data was estimated using Pearson product-moment correlation coefficient. All statistical analyses were performed using R software version 2.12.0 [75].

## Authors' contributions

TTB performed sampling, laboratory work, data analysis, and drafted and revised the manuscript. CFCL participated in sampling and commented on the draft manuscript. BOK and TF were involved in bioinformatics. JMOF and SDJ participated in planning the study and manuscript organization. IB coordinated the study in all stages and revised the manuscript. All authors approved the final manuscript.

## Supplementary Material

Additional file 1 **Read counts for 8 developmental stages of Atlantic halibut**. The total number of raw sequence reads obtained from SOLiD sequencing and the number of usable reads for each developmental stage is given. Click here for file

Additional file 2 **Size distribution of mapped reads in Atlantic halibut deep sequencing data**. Reads mapped to different databases: A) mitochondrial transcripts, rRNA, tRNA, other non-coding RNA; B) miRBase 16 and C) Atlantic halibut ESTs. The size distribution of mapped reads for each database is given. Click here for file

Additional file 3 **Identified conserved miRNAs**. All identified conserved Atlantic halibut miRNAs along with miRBase name, nucleotide sequences and read counts for each investigated developmental stages are given. Lower case letters indicate mismatches comparing to references. Click here for file

Additional file 4 **miRNA diversity during early development of Atlantic halibut**. The number of different types of miRNAs identified during 8 stages of Atlantic halibut development; increasing as the development progressed. Click here for file

Additional file 5 **IsomiR occurrence during early development of Atlantic halibut**. All miRNAs having ≥ 0.01% truncation/s or addition/s in 5' and/or 3' ends are listed. The percentage of both template and non-template nucleotide changes (A, U, C, and G) are shown as percentage of total reads of a given miRNA. Click here for file

Additional file 6 **Cloned miRNA sequences and their frequency**. Indentified conserved miRNAs using cloning technique and their frequencies. Nucleotide mismatches (green), additions (blue) or modifications (red) are found in some miRNAs. Click here for file
