# Supplementary material for: Differential expression patterns of conserved miRNAs and isomiRs during Atlantic halibut development
Source: BMC Genomics. 2012 Jan 10;13:11. doi: 10.1186/1471-2164-13-11 (PMC3398304; doi:10.1186/1471-2164-13-11)
Supplement: Additional file 1 — Read counts for 8 developmental stages of Atlantic halibut. The total number of raw sequence reads obtained from SOLiD sequencing and the number of usable reads for each developmental stage is given. [file 1471-2164-13-11-S1.PDF]

Sample	*Total reads*	**Adaptor removed and >16 nts reads**
Blastula	7 821 764	5 377 768
Epiboly	7 025 828	5 607 336
Somites	10 274 734	7 109 431
Hatching	8 150 274	5 945 983
First feeding	8 770 845	6 609 814
Early metamorphosis	10 047 781	6 778 049
Climax metamorphosis	10 481 279	7 256 435
Juvenile	9 806 331	6 743 576
Total	72 378 836	51 428 392
