# Supplementary figures and images for: Differential expression patterns of conserved miRNAs and isomiRs during Atlantic halibut development
Source: BMC Genomics. 2012 Jan 10;13:11. doi: 10.1186/1471-2164-13-11 (PMC3398304; doi:10.1186/1471-2164-13-11)

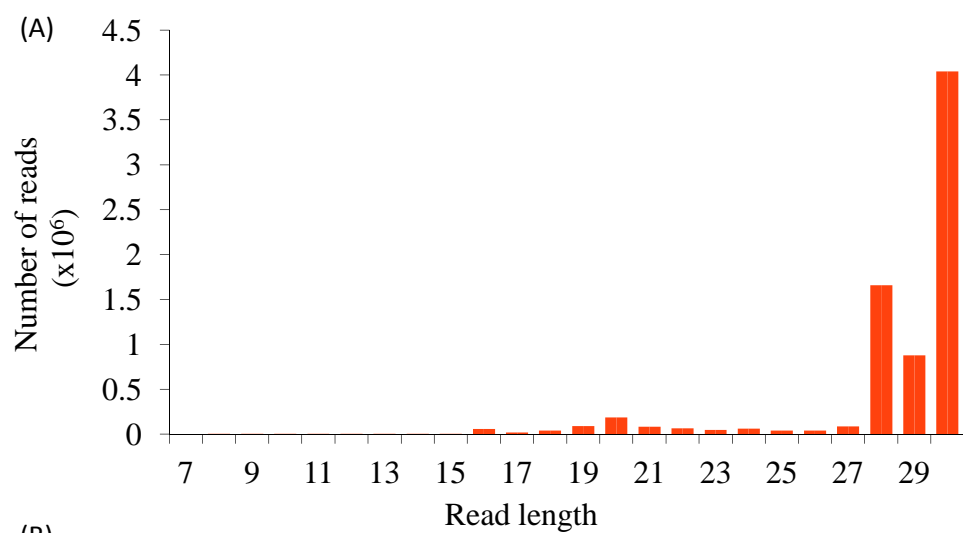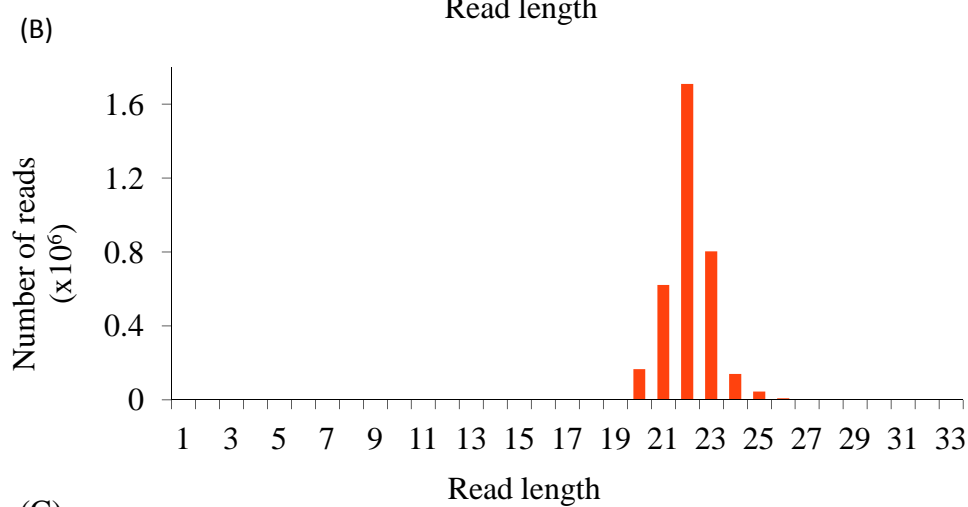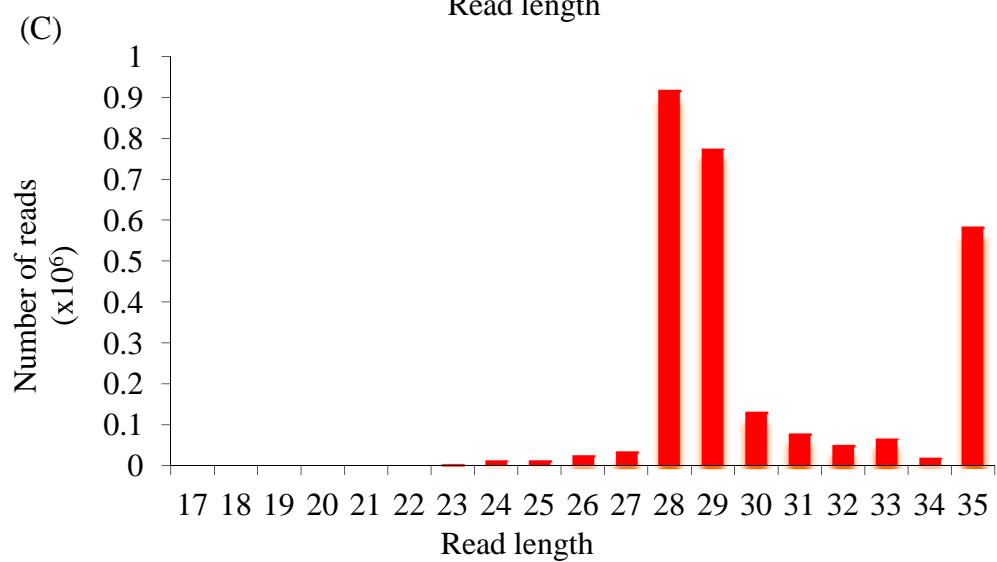

Supplement: Additional file 2 — Size distribution of mapped reads in Atlantic halibut deep sequencing data. Reads mapped to different databases: A) mitochondrial transcripts, rRNA, tRNA, other non-coding RNA; B) miRBase 16 and C) Atlantic halibut ESTs. The size distribution of mapped reads for each database is given. [file 1471-2164-13-11-S2.PDF]

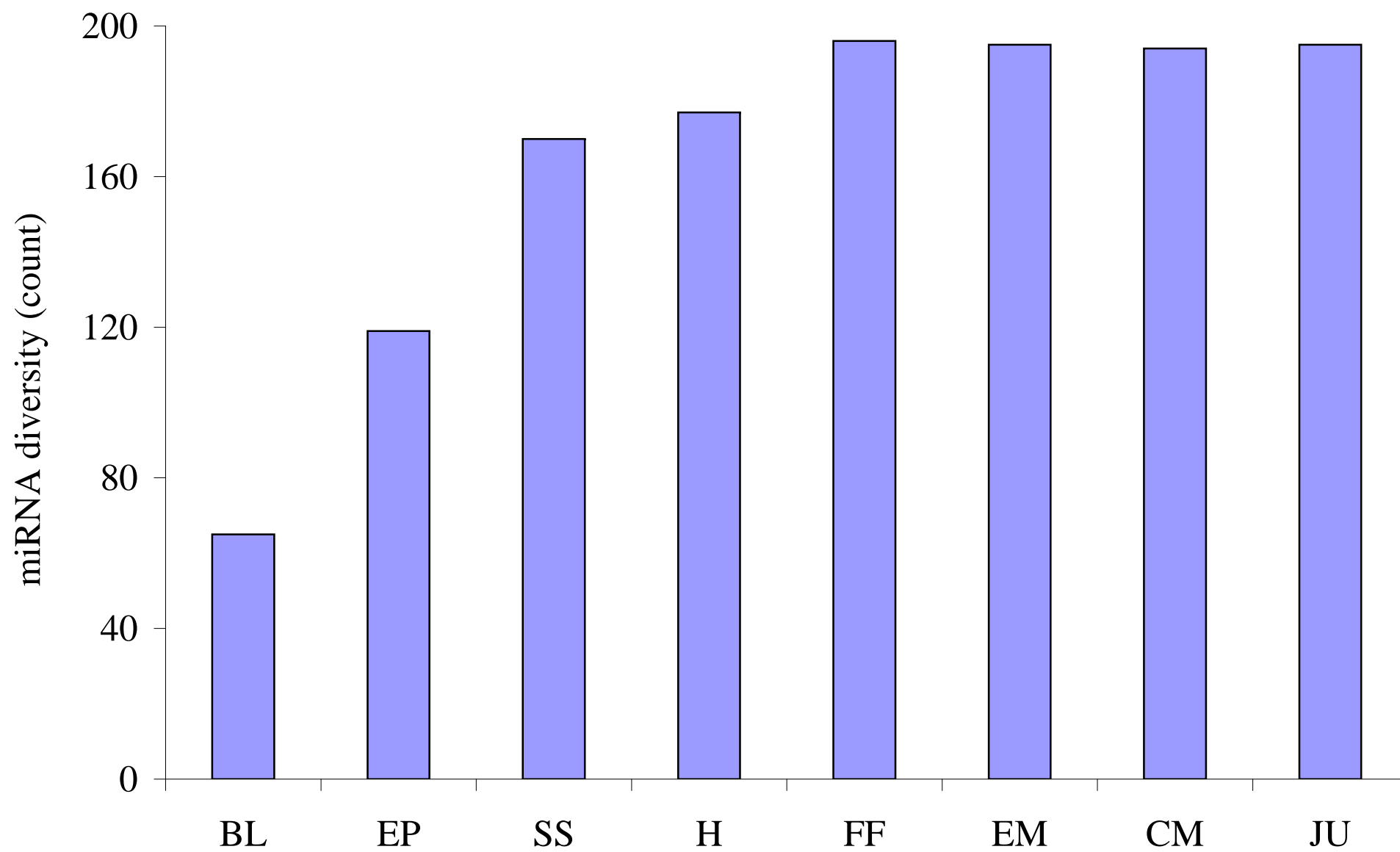

Supplement: Additional file 4 — miRNA diversity during early development of Atlantic halibut. The number of different types of miRNAs identified during 8 stages of Atlantic halibut development; increasing as the development progressed. [file 1471-2164-13-11-S4.PDF]
