# Supplementary material for: Differential expression patterns of conserved miRNAs and isomiRs during Atlantic halibut development
Source: BMC Genomics. 2012 Jan 10;13:11. doi: 10.1186/1471-2164-13-11 (PMC3398304; doi:10.1186/1471-2164-13-11)
Supplement: Additional file 3 — Identified conserved miRNAs. All identified conserved Atlantic halibut miRNAs along with miRBase name, nucleotide sequences and read counts for each investigated developmental stages are given. Lower case letters indicate mismatches comparing to references. [file 1471-2164-13-11-S3.PDF]

miRNAs	Sequence	BL	EP	SS	HA	FF	EM	CM	JU
let-7a	UGAGGUAGUAGGUUGUAUAGUU	3	64	44	64	1888	2612	2041	2107
let-7b	UGAGGUAGUAGGUUGUGUGGUU	4	14	6	2	105	238	226	323
let-7c	UGAGGUAGUAGGUUGUAUGGUU	6	19	20	16	383	396	270	311
let-7d	UGAGGUAGUUGGUUGUAUGGUU	2	2	2	6	1048	1283	611	642
let-7e	UGAGGUAGUAGAUUGAAUAGUU	1	9	1	2	163	354	367	428
let-7f	UGAGGUAGUAGAUUGUAUAGUU	1	4	1	1	43	281	261	324
let-7g	UGAGGUAGUAGUUUGUAUAGUU	6	10	36	41	668	973	751	754
let-7h	UGAGGUAGUAAGUUGUGUUGUU	0	0	0	1	50	180	149	230
let-7i	UGAGGUAGUAGUUUGUGCUG	0	10	6	8	324	488	413	485
let-7j	UGAGGUAGUUGUUUGUACAGUU	5	7	18	20	836	1618	949	1160
miR-1	UGGAAUGUAAAGAAGUAUGUAU	10	9	2891	19587	35624	33792	32242	32339
miR-100	AACCCGUAGAUCCGAACUUGUG	10	61	54	315	4119	2577	2534	2570
miR-101a	UACAGUACUGUGAUAACUGAAG	0	2	4	9	354	218	121	106
miR-101b	GUACAGUACUAUGAUAACUGAA	0	11	73	124	772	724	461	386
miR-103	AGCAGCAUUGUACAGGGCUAUGA	15	61	2350	4185	25871	8274	5542	7265
miR-107	AGCAGCAUUGUACAGGGCUAUCA	3	43	922	1536	6439	2655	1965	2527
miR-107b	AGCAGCAUUGUACAGGGCUUUCAG	1	2	52	106	539	166	103	149
miR-10a	UACCCUGUAGAUCCGAAUUUGU	1	2	12	16	31	8	3	4
miR-10b-1	UACCCUGUAGAACCGAAUUUGU	6	5	2237	2953	4999	1240	1146	930
miR-10c	UACCCUGUAGAUCCGGAUUUGU	5	3	1034	1667	3091	974	994	761
miR-10d	UACCCUGUAGAACCGAAUGUGU	4	8	5276	6530	10600	2155	1695	1411
miR-122	UGGAGUGUGACAAUGGUGUUUG	2	6	64	95	1938	3451	4321	4681
miR-124	UAAGGCACGCGGUGAAUGCCA	24	259	827	3320	37905	12190	6313	5640
miR-125a	UCCCUGAGACCCUUAACCUGUGA	14	45	44	165	8576	4576	4302	3922
miR-125b	UCCCUGAGACCCUAACUUGUGA	25	142	175	1018	17171	8820	8172	7781
miR-125c	UCCCUGAGACCCUAACUCGUGA	3	16	25	86	1722	808	939	639
miR-126	UCGUACCGUGAGUAAUAAUG	5	8	314	564	3002	2641	2144	2310
miR-126*	CAUUAUUACUUUUGGUACGCG	2	9	424	915	2373	2195	1633	1898
miR-126b	UCGUACCGUGAGUAAUAGUGCA	0	4	71	124	769	472	461	397
miR-128	UCACAGUGAACCGGUCUCUUU	5	39	192	302	1378	619	670	480
miR-129	CUUUUUGCGGUCUGGGCUUG	0	2	5	20	558	86	88	76
miR-129*	AAGCCCUUACCCCAAAAAGCAU	5	46	34	60	2427	776	618	471
miR-130a	CAGUGCAAUGUUAAAAGGGCAU	0	4	12	48	123	65	17	67
miR-130b	CAGUGCAAUAAUGAAAGGGCAU	6	81	3020	7933	12362	5699	2893	5316
miR-130c	CAGUGCAAUAUUAAAAGGGCAU	148	3173	27830	77044	93055	51054	20644	42077
miR-132	UAACAGUCUACAGCCAUGGU	1	8	7	17	633	134	114	73
miR-132*	ACCGUGGCAUUAGAUUGUUACU	0	0	0	0	47	28	9	22
miR-133a	UUUGGUCCCCUUCAACCAGCU	0	0	416	2009	2419	3363	3735	3673
miR-133a*	AGCUGGUAAAAUGGAACCAAA	5	4	3647	17469	4826	12185	11944	13509
miR-133b	UUUGGUCCCCUUCAACCAGCU	1	5	1891	2632	2283	2870	2754	2355
miR-133b*	GGCUGGUCAAAUGGAACCAA	0	38	1846	1040	185	246	152	226
miR-133c	UUUGGUCCCUUUCAACCAGCUA	0	0	0	5	5	7	3	2
miR-135a	UAUGGCUUUUUAUUCCUAUGUGA	1	13	44	112	2378	511	419	341
miR-135b	UAUGGCUUUUUAUUCCUAUCUG	0	2	17	41	617	125	97	83
miR-135c	UAUGGCUUUCUAUUCCUAUGUGA	0	7	46	68	2655	717	538	389
miR-137	AUUGCUUAAGAAUACGCGUAG	0	1	13	70	1047	290	201	175
miR-138	AGCUGGUGUUGUGAAUCAGGCCG	12	54	452	1271	10462	4482	3894	3386
miR-1388	AUCUCAGGUUCGUCAGCCCAUG	0	0	1	6	16	41	34	46
miR-1388*	cGGACUGUCCAACCUGAGAAUG	0	0	2	1	3	9	3	7
miR-139	UCUACAGUGCAUGUGUCUCCAGU	1	2	484	374	534	172	169	111
miR-140	CAGUGGUUUUACCCUAUGGUAG	3	0	95	300	2907	3545	2272	1987
miR-140*	UACCACAGGGUAGAACCACGGAC	6	21	694	923	9349	7765	6087	6772
miR-141	UAACACUGUCUGGUAACGAUGC	0	49	255	410	706	797	647	586
miR-142a-3p	UGUAGUGUUUCCUACUUUAUGG	0	0	0	7	42	241	75	140
miR-142b-5p	GUAGACAGCACUACUAAACU	0	0	0	1	2	43	9	32

miR-143	UGAGAUGAAGCACUGUAGCUC	2	7	46	123	1073	1778	1129	1436
miR-144	CUACAGUAUAGAUGAUGUACUA	0	0	0	0	0	8	10	9
miR-145	GUCCAGUUUUCCCAGGAAUCCCU	58	64	348	494	4267	4657	5053	4543
miR-146a	UGAGAACUGAAUUCCAUAGAUGG	1	6	41	49	413	1677	1097	1191
miR-146b	UGAGAACUGAAUUCCAAGGGUG	0	0	0	0	2	36	19	34
miR-148	UCAGUGCAUUACAGAACUUUG	3	8	1023	1916	3876	5121	3852	3130
miR-150	aCUCCCAAUCCUUGUACCAGUGU	0	1	1	0	3	209	213	231
miR-152	UCAGUGCAUaACAGAACUUUG	1	4	92	214	401	569	440	411
miR-153a	UUGCAUAGUCACAAAAGUGAUCAU	0	6	29	152	903	425	274	250
miR-153b	UUGCAUAGUCACAAAAAUGAGCA	0	0	4	24	253	105	75	82
miR-153c	UUGCAUAGUCACAAAAAUGAUCA	0	0	10	26	77	30	33	21
miR-15a	UAGCAGCACgGAAUGGUUUGU	6	34	301	427	1404	1030	614	592
miR-15b	UAGCAGCGCAUCAUGGUUUGaA	19	135	968	1229	2518	1816	1570	1174
miR-16a	UAGCAGCACGUAAAUAUUGG	4	13	94	98	261	146	122	133
miR-16b	UAGCAGCACGUAAAUAUUGGAG	18	132	855	1091	1811	1017	803	847
miR-16c	UAGCAGCAUGUAAAUAUUGGAG	0	0	0	1	5	0	1	0
miR-1788	GGCUUGUUUUAAGUUGCCUGCG	1	0	34	44	86	81	58	98
miR-1788*	CAGGCAGCUAAAGCAAGUCU	0	0	51	90	380	497	444	423
miR-17a	CAAAGUGCUUACAGUGCAGGUA	55	2427	11300	16083	24420	17223	11956	12803
miR-17a*	ACUGCAGUGGAGGCACUUacAGCAgU	1	62	32	25	13	6	7	4
miR-181a	AACAUUCAACGCUGUCGGUGAGUUU	9	50	1890	3994	20104	8589	6643	6166
miR-181a*	ACCAUCGACCGUUGAUUGUACC	8	125	2079	3527	10051	7037	4964	6020
miR-181b	AACAUUCAUUGCUGUCGGUGGGUU	1	8	1384	3210	7088	3593	2860	2685
miR-181c	aACAUUCAUUGCUGUCGGUGGGUUUU	0	0	16	106	91	26	38	42
miR-182	UUUGGCAAUGGUAGAACUCACACU	1	2	121	223	2822	478	463	373
miR-182*	UGGUUCUAGACUUGCCAACUA	0	0	18	40	1018	230	144	96
miR-183	UAUGGCACUGGUAGAAUUCACUG	0	1	146	237	3274	630	479	425
miR-184	UGGACGGAGAACUGAUAAGGGC	0	4	2330	2550	2010	859	606	875
miR-187	UCGUGUCUUGUGUUGCAGCCAGU	0	15	37	70	452	142	103	104
miR-18a	UAAGGUGCAUCUAGUGCAGAUAG	36	408	2972	4838	5946	4408	2707	2630
miR-18b	UAAGGUGCAUUUAGUGCAGAUAG	0	3	1	8	4	1	0	0
miR-18c	UAAGGUGCAUCUaGUGUAGUUA	0	7	209	378	401	66	36	33
miR-190	UGAUAUGUUUGAUAUAUUAGGUUG	0	1	72	129	364	205	150	130
miR-190b	UGAUAUGUUUGAUAUUCaGUU	0	1	13	15	1262	190	111	132
miR-192	AUGACCUAUGAAUUGACAGCC	13	18	466	1641	23915	41532	35340	42588
miR-193a	AACUGGCCUACAAAGUCCCA	0	0	4	20	245	277	207	189
miR-194a	UGUAACAGCAACUCCAUGUGGAA	0	1	36	108	972	2569	1431	1632
miR-194b	UGUAACAGCauCUCCAUGUGGAA	0	0	0	0	1	16	7	8
miR-196a-1	UAGGUAGUUUCAUGUUGUUGGG	0	0	452	346	482	280	173	205
miR-196b	UAGGUAGUUUCAAGUUGUUGGG	0	1	131	81	68	22	20	13
miR-199	CCCAGUGUUCAGACUACCUGU	9	28	382	1951	30290	43557	29483	30982
miR-199*	UACAGUAGUCUGCACAUUGGUUA	1	17	483	1319	12950	15943	11195	8650
miR-19a	UUGUGCAAAUCUAUGCAAAACUG	12	559	3371	6574	14359	10542	5414	5212
miR-19a*	AGUUUUGCAUAGUUGCACUA	0	120	114	215	120	76	40	58
miR-19b	UGUGCAAAUCCAUGCAAAACUG	137	4345	14791	25100	52358	33731	17202	19109
miR-19b*	AGUUUUGCUGGUUUGCAUUCAG	1	42	16	14	38	9	3	9
miR-19c	UGUGCAAAUCCAUGCAAAACUCG	9	146	512	973	2538	1376	764	861
miR-19d	UGUGCAAACCCAUGCAAAACUGA	112	703	6320	11299	18300	9979	6845	7154
miR-200a	UAACACUGUCUGGUAACGAUGU	2	105	762	1350	3631	3684	2686	2864
miR-200b	UAAUACUGCCUGGUAAUGAUGAU	0	55	1229	1230	1878	1096	1015	1077
miR-200c	UAAUACUGCCUGGUAAUGAUGCA	0	2	54	53	69	45	39	37
miR-202*	UUCCUAUGCAUAUACCUCUUU	2	17	3	1	6	7	6	6
miR-203a	UGUGAAAUGUUUAGGACCACUUG	26	2793	26072	28459	30369	11762	13055	12570
miR-203b	GUGAAAUGUUCAGGACCACUUG	0	0	3	2	6	2	1	2
miR-203b*	AGUGGUUCUCAACAGUUCAACA	3	1467	2710	1875	1987	1188	1036	926
miR-204	UUUCCCUUUGUCAUCCUAUGCCU	4	12	361	970	9854	3533	3008	2256

miR-205	UCCUUCAUUCCACCGGAGUCUG	15	259	18761	17721	11936	6328	10351	7229
miR-206	UGGAAUGUAAGGAAGUGUGUGG	6	40	13201	20602	15572	24216	15016	21359
miR-20a	UAAAGUGCUUAUAGUGCAGGUAG	18	798	3468	5911	6760	4428	3082	2978
miR-20a*	ACUGCAGUGUGAGCACUUGAAGU	0	214	96	53	129	57	43	40
miR-20b	CAAAGUGCUCACAGUGCAGGUA	1	29	449	709	568	147	155	212
miR-21	UAGCUUAUCAGACUGGUGUUGGC	15	287	1830	1252	7790	6044	5537	3552
miR-210	CUGUGCGUGUGACAGCGGCUAACC	2	13	27	72	2481	6850	5447	5932
miR-210*	AGCCACUGACUAACGCACAUUG	2	19	68	117	1420	3201	3480	4073
miR-212	UAACAGUCUACAGUCAUGGCUAC	0	4	0	4	387	104	55	47
miR-214	UACAGCAGGCACAGACAGGCA	0	3	113	547	4317	4759	3302	4812
miR-216a	UAAUCUCAGCUGGCAACUGUGAG	0	0	5	34	91	76	73	74
miR-216b	UAAUCUCUGCAGGCAACUGUGA	1	7	41	183	1033	458	187	217
miR-217	UACUGCAUCAGGAACUGAUUGG	15	13	43	115	1167	505	386	351
miR-2184	AACAGUAAGAGUUUAUGUGCUG	0	2	1	0	293	465	321	381
miR-2188	AAGGUCCAACCUCACAUGUCCU	2	11	112	204	3892	838	705	575
miR-218a	UUGUGCUUGAUCUAACCAUGUG	4	16	768	1310	7739	2446	2282	1997
miR-218b	UUGUGCUUGAUCUAACCAUGCA	2	4	195	608	8810	1942	1211	1193
miR-219	UGAUUGUCCAAACGCAAUUCUU	0	3	11	84	62	103	40	44
miR-221	ACCUGGCAUACAAUGUAGAUU	1	6	806	1133	2638	949	961	640
miR-222	AGCUACAUCUGGCUACUGGGUCUC	1	6	975	1413	2111	579	648	583
miR-223	UGUCAGUUUGUCAAAUACCCCAA	3	9	73	226	834	997	1180	1389
miR-22a	AAGCUGCCAGCUGAAGAACUGU	35	55	1993	4505	15887	18675	12814	13107
miR-22b	AAGCUGCCAGUUGAAGAGCU	0	0	8	33	134	139	78	91
miR-23a	AUCACAUUGCCAGGGAUUUCCA	3	1	249	371	1506	1966	2365	2351
miR-23b	AUCACAUUGCCAGGGAUUACCAC	3	6	184	340	2328	1751	1741	1723
miR-24	UGGCUCAGUUCAGCAGGAACAG	22	54	2374	4144	16838	17463	14746	19793
miR-25	CAUUGCACUUGUCUCGGUCUGA	44	240	1337	1776	3582	1917	1504	1310
miR-26a	UUCAAGUAAUCCAGGAUAGGCU	17	135	1132	1581	8170	3507	2760	2440
miR-26b	UUCAAGUAAUCCAGGAUAGGUU	7	39	237	393	1434	785	684	562
miR-27a	UUCACAGUGGCUAAGUUCCG	0	1	5	6	153	146	115	134
miR-27b	UUCACAGUGGCUAAGUUCUGCA	1	2	35	59	523	311	262	269
miR-27c	UUCACAGUGGUUAAGUUCUGCC	0	1	23	81	450	823	670	673
miR-27d	UUCACAGUGGCUAAGUUCUUCAC	0	1	6	8	72	58	39	30
miR-27e	UUCACAGUGGCUAAGUUCAGU	0	1	52	80	355	345	356	359
miR-29a	UAGCACCAUUaGAAAUCGGUUA	9	57	51	188	3422	749	545	480
miR-29b	UAGCACCAUUUGAAAUCAGUGU	14	26	9	29	834	439	256	244
miR-301a	CAGUGCAAUAGUAUUGUCAAAGCG	0	9	456	1016	1781	1338	852	1021
miR-301b	CAGUGCAAUAGUAUUGUCAUUGC	0	1	251	657	1015	560	355	368
miR-301c	CAGUGCAAUAGUAUUGUCAUAGC	5	66	5286	14545	27542	16177	10343	11414
miR-30b	UGUAAACAUCCUACACUCAGCU	3	17	510	789	2040	1435	1087	937
miR-30c	UGUAAACAUCCUACACUCUCAGCU	5	69	1378	1673	3266	1622	1316	1088
miR-30d	UGUAAACAUCCCCGACUGGAAG	5	8	311	637	1436	799	803	770
miR-30e	UGUAAACAUCCUUGACUGGAAGC	5	75	505	1302	2836	3540	1932	2313
miR-30e*	CUUUCAGUCGGAUGUUUGCAGC	1	109	1324	1114	1295	388	555	525
miR-31	aGGCAAGAUGUUGGCAUAGCUG	0	1	10	22	11	4	10	23
miR-338	UCCAGCAUCAGUGAUUUUGUUGC	2	26	34	228	4687	5051	3557	3666
miR-34	UGGCAGUGUCUUAGCUGGUUGU	2	103	253	870	4814	2060	1477	1841
miR-34b	AGGCAGUGUUGUUAGCUGAUUG	0	0	0	3	16	6	11	12
miR-363	AAUUGCACaGUAUCCAUCUG	0	5	17	37	56	21	13	6
miR-365	UAAUGCCCCUAAAAAUCCUUAU	1	0	7	38	169	171	206	144
miR-375	UUUGUUCGUUCGGCUCGCGUUA	0	0	412	457	1287	154	346	277
miR-429	UAAUACUGUCUGGUAAUGCCGU	0	18	584	584	805	582	451	469
miR-429b	UAAUACUGCCUGGUAAUGCC	0	0	24	17	40	29	31	30
miR-430a	UAAGUGCUAUUUGUUGGGGUAG	270	4371	4282	2625	53	0	0	0
miR-430c	UAAGUGCUUCUCUUUGGGGUAG	2906	73401	22495	14671	97	4	6	13

miR-430i	UAAGUGCUAUUUGUUGGCGUAG	21	306	187	111	2	0	0	0
miR-451	AAACCGUUACCAUUACUGAGUUU	2	9	0	1	7	134	114	170
miR-454a	UAGUGCAAUAUUGCUAAUAGGG	0	0	0	1	5	2	2	0
miR-454b	AGUGCAAUAUUGCUUAUAGGGUCU	0	0	21	55	103	63	33	31
miR-455	AUGCAGUCCAUGGGCAUAUACA	0	0	23	85	668	778	469	444
miR-455b	UAUGUGCCCUUGGACUACAUUG	0	0	9	30	120	147	89	96
miR-456	CAGGCUGGUUAGAUGGUUGUC	1	5	94	107	408	267	238	231
miR-458	AUAGCUCUUUGAAUGGUACUG	3	5	110	145	228	478	370	391
miR-459	UCAGUAACAAGGAUUCAUCCUG	0	1	0	0	346	1048	500	600
miR-460-3p	CACAGCGCAUACAAUGUGGAU	0	0	1	4	31	45	45	48
miR-460-5p	CCUGCAUUGUACACACUGUGCG	0	3	11	88	534	1011	935	920
miR-462	GUAACGGAACCCAUAAUGCAGCU	0	5	5	14	21	21	31	21
miR-489	UGACAUCAUAUGUACGaCUGCU	0	6	0	1	90	47	50	41
miR-499	UUAAGACUUGCAGUGAUGUUUA	0	0	45	103	333	228	199	90
miR-722	UUUUGCAGAAACGUUUCAGAUU	0	0	1	2	13	9	16	15
miR-724	UAAAGGGAAUUUGCGACUGUU	0	2	3	4	229	49	31	36
miR-725	UUCAGUCAUUGUUUCUAGUAG	0	6	203	230	145	81	89	58
miR-727	GUUGAGGCGAGUUGAAGACUU	1	2	14	30	69	24	13	10
miR-727*	UCAGUCUUCAAUUCCUCCCAGC	0	2	128	70	168	29	37	17
miR-728	AUACUAAGUACACUACGUUUU	0	0	34	39	44	13	5	6
miR-729	CAUGGGUAUGAUACGACCUG	0	0	0	0	10	9	14	14
miR-730	UCCUCAUUGUGCAUGCUGUGUG	1	8	51	87	602	159	170	205
miR-731	AAUGACACGUUUUCUCCCGGAUCGC	0	0	12	16	34	27	23	28
miR-733	GCGUUGGUUUAGCUCAGUGGUUACUU	2	1	7	9	12	41	12	12
miR-737	AAAUCAAAACCUAAAGAAAAU	0	0	0	3	6	8	3	1
miR-7a	UGGAAGACUAGUGAUUUUGUUGU	1	32	151	337	2468	838	569	663
miR-9	UCUUUGGUUAUCUAGCUGUAUGA	4	69	250	978	28885	4804	3386	2886
miR-9*	AUAAAGCUAGAUAACCGAAAGU	1	11	38	187	3454	892	542	428
miR-92a	UAUUGCACUUGUCCCGGCCUGU	25	1409	4909	4162	5524	2346	1881	1664
miR-92b	UAUUGCACUCGUCCCGGCCUCC	0	0	15	3	8	29	22	27
miR-93	AAAAGUGCUGUUUGUGCAGGUAG	6	133	1190	2521	2531	1465	1226	1049
miR-96	UUUGGCACUAGCACAUUUUUGCU	0	0	6	13	381	96	37	38
miR-99	AACCCGUAGAUCCGAUCUUGUG	8	41	143	733	5625	2099	1953	1835

1
